# Week-Old Chicks with High *Bacteroides* Abundance Have Increased Short-Chain Fatty Acids and Reduced Markers of Gut Inflammation

**DOI:** 10.1128/spectrum.03616-22

**Published:** 2023-01-31

**Authors:** Yi Fan, Tingting Ju, Tulika Bhardwaj, Douglas R. Korver, Benjamin P. Willing

**Affiliations:** a Department of Agricultural, Food and Nutritional Science, University of Alberta, Edmonton, Alberta, Canada; Chengdu University

**Keywords:** broiler chickens, *Bacteroides*, gut microbiota, microbial functionality, inflammation

## Abstract

As important commensals in the chicken intestine, *Bacteroides* are essential complex carbohydrate degraders, and short-chain fatty acid (SCFA) producers that are highly adapted to the distal gut. Previous studies have shown large variation in *Bacteroides* abundance in young chickens. However, limited information is available regarding how this variation affects the gut microbiome and host immunity. To investigate how elevated or depleted *Bacteroides* levels affect gut microbial functional capacity and impact host response, we sampled 7-day-old broiler chickens from 14 commercial production flocks. Week-old broiler chickens were screened and birds with low *Bacteroides* (LB) and high *Bacteroides* (HB) abundance were identified via 16S rRNA gene amplicon sequencing and quantitative PCR (qPCR) assays. Cecal microbial functionality and SCFA concentration of chickens with distinct cecal *Bacteroides* abundance were profiled by shotgun metagenomic sequencing and gas chromatography, respectively. The intestinal immune responses of LB and HB chickens were assessed via reverse transcription qPCR. Results showed that the gut microbiota of the LB group had increased abundance of lactic acid bacteria pyruvate fermentation pathway, whereas complex polysaccharide degradation and SCFA production pathways were enriched in the HB group (*P < *0.05), which was supported by increased SCFA concentrations in the ceca of HB chickens (*P < *0.05). HB chickens also showed decreased expression of *interleukin-1β* and increased expression of *interleukin-10* and tight-junction protein *claudin-1* (*P < *0.05). Overall, the results indicated that elevated *Bacteroides* may benefit the 7-day broiler gut and that further work should be done to confirm the causal role of *Bacteroides* in the observed positive outcomes.

**IMPORTANCE** To date, limited information is available comparing distinct *Bacteroides* compositions in the chicken gut microbial communities, particularly in the context of microbial functional capacities and host responses. This study showed that possessing a microbiome with elevated *Bacteroides* in early life may confer beneficial effects to the chicken host, particularly in improving SCFA production and gut health. This study is among the first metagenomic studies focusing on the early life chicken gut microbiota structure, microbial functionality, and host immune responses. We believe that it will offer insights to future studies on broiler gut microbial population and their effects on host health.

## INTRODUCTION

In the ceca of matured chickens, *Firmicutes* and *Bacteroidetes* are reported to be the most dominant phyla, where together these 2 phyla represent more than 90% of total cecal microbiota (1–3). It has been shown that *Bacteroides* have relatively low abundance in the ceca of newly hatched chicks (4), and become the predominant taxa at day 7, reaching the peak (40 to 45%) at 3 weeks of age (5). Great variation of *Bacteroides* abundance was reported in the ceca of young chickens ranging from 2% to 40% (6, 7). Members belonging to the genus *Bacteroides* are Gram-negative, rod-shaped bacteria, which are highly adapted to the gut environment, especially the lower gastrointestinal tract. Encoding a high number of genes for polysaccharide and monosaccharide metabolism, *Bacteroides* are important complex carbohydrates degraders in the host gut (8). However, limited information is available regarding how differential abundance of this taxa affects gut immune state or functional capacity of the gut microbiota in broiler chickens.

In microbiome research, studying variations in microbial structure and composition can offer insight into complex host-microbe-metabolite interactions. Arumugam et al. (2011) first described 3 robust clusters in the human gut microbiota, indicating the importance of the population-level analysis of the gut microbiome variation (9). In chicken research, studies have also suggested the existence of distinct gut microbiomes among individuals (10, 11). Kaakoush et al. (2014) reported that chicken fecal microbiomes could be separated into 4 enterotypes, including elevated *Bacteroides*, and that microbial composition could be associated with pathogen carriage; however, the authors did not explore changes in metabolite profile or host responses (10). A more recent study identified high *Bacteroides* in the duodenum of mature chickens with less fat deposition, and lower serum triglyceride levels (11).

The aim of this study was to understand how high and low *Bacteroides* abundances are associated with early life chicken gut microbial functional capacity, and immune response. This was achieved by sampling and characterizing week-old broiler chickens from commercial production flocks with distinct cecal *Bacteroides* abundance.

## RESULTS

### Bacterial composition of early life chicken cecal microbiome.

On average, 24,647.56 ± 7632.78 reads per sample were generated, and processed by the QIIME2 pipeline, resulting in 1,798 amplicon sequence variants (ASVs). Filtered reads were taxonomically classified to represent 4 major phyla (Firmicutes, Bacteroidetes, Proteobacteria, and Actinobacteria) and 106 genera. The 3 most abundant phyla made up over 98% of the population, and included Firmicutes (76.16 ± 15.72%), Bacteroidetes (17.54 ± 16.59%), and Proteobacteria (5.07 ± 6.83%).

### *Bacteroides* over-/under- representing sample identification.

On the genus level, various levels of *Bacteroides* relative abundance were observed (16.3 ± 15.6%; range, 0 to 52.2%). Low *Bacteroides* samples were defined as samples with *Bacteroides* relative abundance lower than 0.7% (mean − SD); whereas high *Bacteroides* samples were defined as samples with *Bacteroides* relative abundance higher than 31.9% (mean + SD). As a result, chickens from 11 different flocks were assigned to either the LB or HB group. Specifically, 18 birds with low *Bacteroides* levels from 6 flocks and 15 birds with high *Bacteroides* from 6 flocks were identified. Chickens that were not assigned to either group were marked as not assigned (n/a) (Table 1). Bodyweights were not collected at day 7 terminations; however, the average 32-day flock bodyweight (*P = *0.91) and mortality rate (*P = *0.93) were similar between flocks that had the majority of birds identified as LB, HB, or n/a (Table 1).

**TABLE 1:** Distribution of chickens assigned to high *Bacteroides* group, low *Bacteroides* group, or unassigned^*a*^

Chicken distribution	Flock mean bodyweight (gram)	Flock mean mortality rate (%)
HB^*b*^ (1) + n/a (4)	1,793	4.8
LB^*c*^ (5)	1,746	4.7
LB (4) + n/a (1)	1,810	3.3
LB (4) + HB (1)	1,836	5.7
LB (2) + n/a (3)	1,790	6.5
LB (1) + n/a (4)	1,850	3.1
HB (1) + n/a (4)	1,798	4.8
n/a^*d*^ (5)	1,744	5.2
HB (1) + n/a (4)	1,750	5.1
HB (3) + n/a (2)	1,762	7.0
n/a (5)	1,850	6.1
HB (3) + n/a (2)	1,886	3.7
HB (3) + n/a (2)	1,790	3.5
n/a (5)	1,744	5.2

a Each box represents a single production flock. In each production flock, 5 young broiler chickens were randomly sampled. Numbers in the brackets refer to the number of broiler chickens assigned to each specific group.

b HB, high *Bacteroides* group.

c LB, low *Bacteroides* group.

d n/a, unassigned.

**FIG 1:**
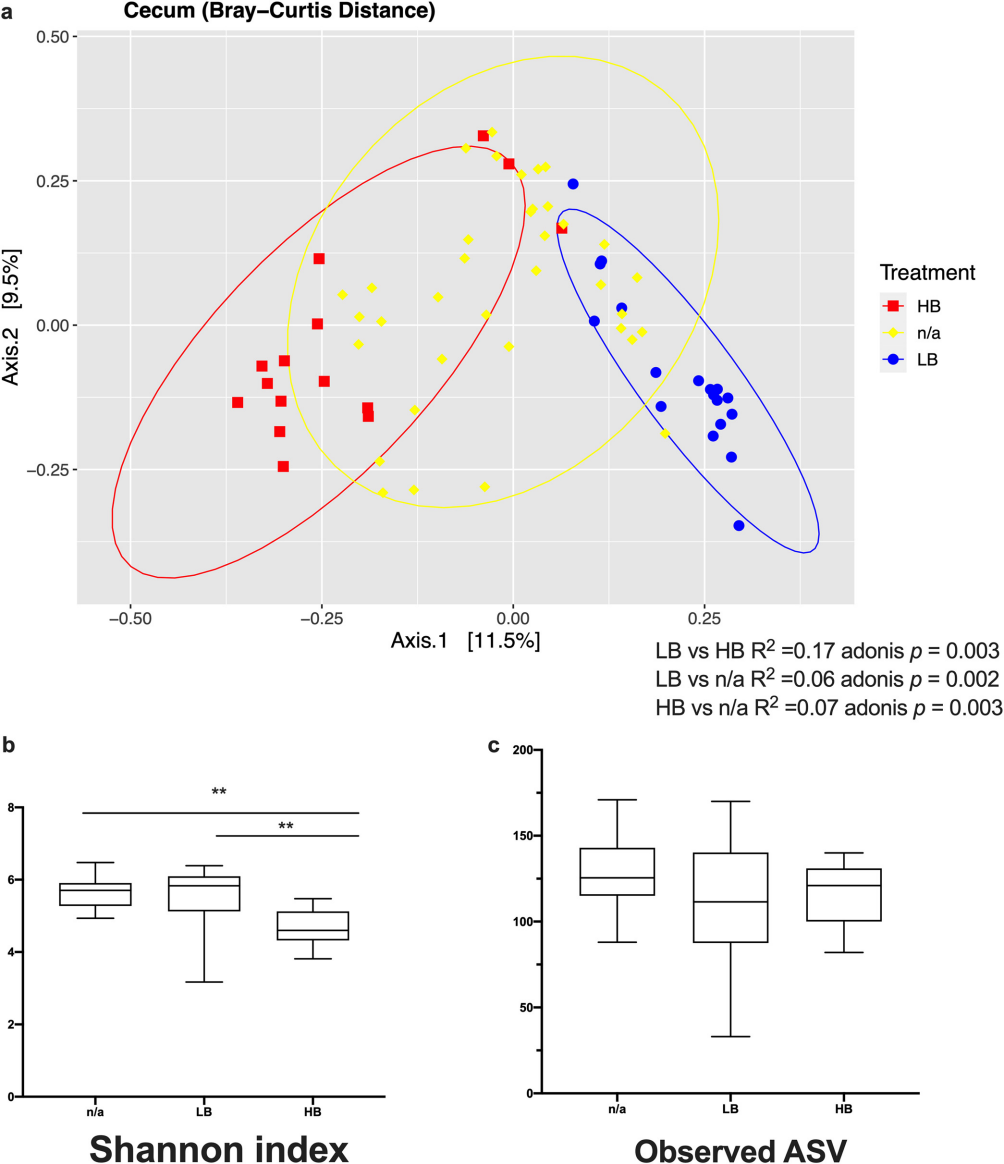
(a) Principal coordinate analysis plot based on Bray-Curtis distance metric. Distinct pattern of Bacteroides populations resulted in significantly different clusters between LB, HB, and chickens not assigned to either HB or LB groups (n/a) (adonis *P* < 0.05). (b) The HB group had decreased bacterial species evenness compared with the LB group and the n/a group (Shannon index, *P* < 0.01). (c) Comparable bacterial species richness was observed between groups (observed ASV, *P* > 0.05). LB, low Bacteroides, *n* = 18; HB, high Bacteroides, *n* = 15; n/a, not assigned, *n* = 37. *, *P* < 0.05; **, *P* < 0.01.

Beta diversity analyses revealed that LB, HB, and n/a groups were significantly separated based on Bray-Curtis distance metric (*P < *0.05 for HB versus LB, HB versus n/a, and LB versus n/a) (Fig. 1). Observed ASV index revealed that the richness of observed taxa between the HB, LB, and n/a groups was comparable. However, the Shannon index showed that the HB had decreased evenness due to the high relative abundance of *Bacteroides* in the cecal microbiota. Quantitative PCR (qPCR) assay using primers targeting the *Bacteroides-Prevotella* group showed that the LB group had a lower absolute abundance of *Bacteroides-Prevotella* group (5.96 and 9.04 log_10_ copies/g cecal contents of *Bacteroides-Prevotella* 16S rRNA gene for LB and HB group, respectively; *P < *0.01).

**FIG 2:**
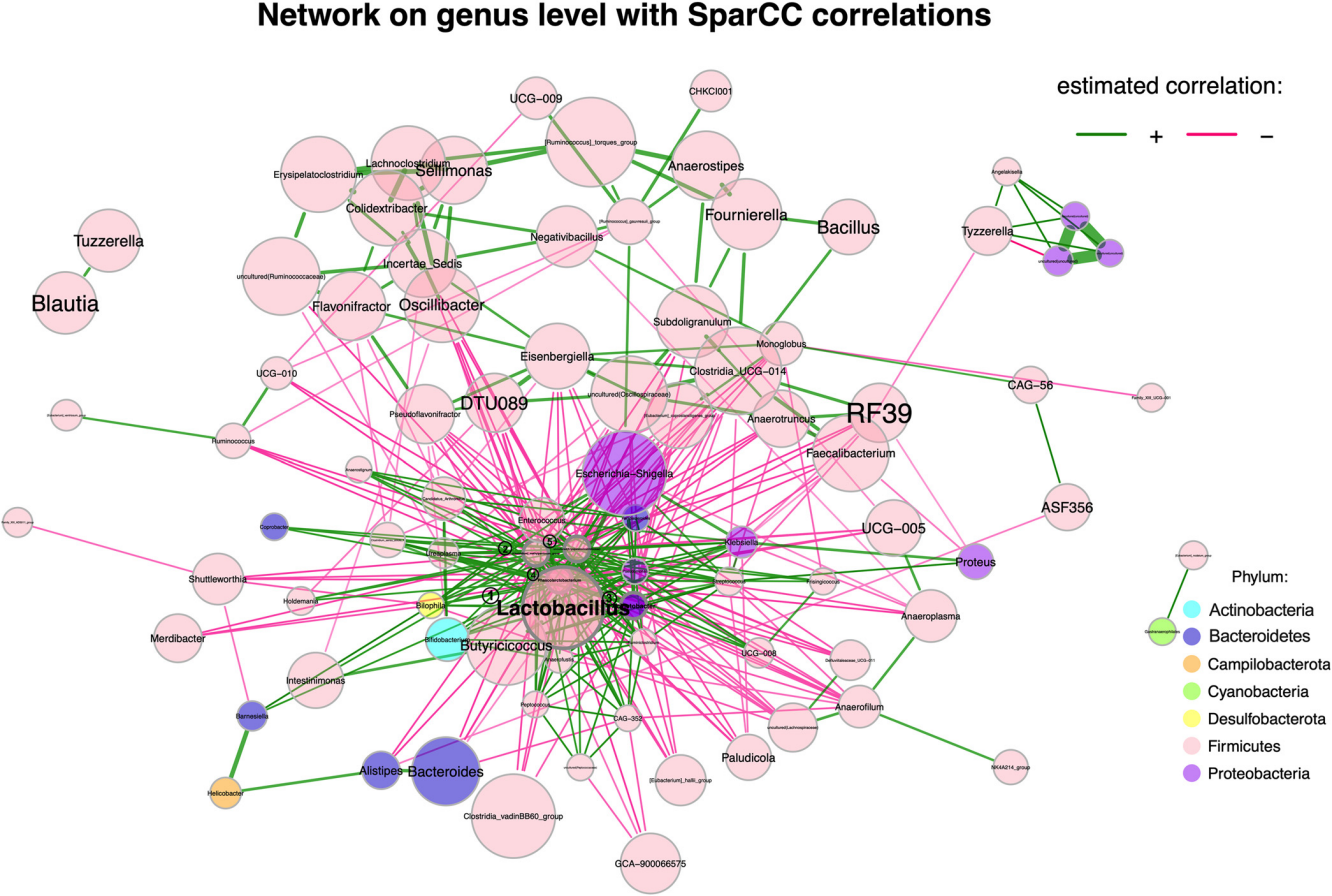
Cecal microbiome co-occurrence network based on SparCC correlation on the genus level. Only significantly correlated (*P < *0.05) taxa in the cecal microbiome with coefficient greater than 0.3 are shown. Estimated correlations were transformed to dissimilarities via the “signed” distance metric, and corresponding similarities were used as edge weights. Node sizes were scaled based on eigenvector centrality. Hubs were defined using eigenvector centrality with a centrality value above the empirical 90% quantile. To increase visibility, hubs were highlighted by bold text and borders, and marked as ①, ②, ③, ④, and ⑤. Node colors represent phyla. ①, *Lactobacillus*; ②, *[Clostridium]_methylpentosum_group;* ③, Acinetobacter; ④, *Phascolarctobacterium*; ⑤, an uncultured member from the family *Erysipelatoclostridiacea*.

The generated cecal microbiome network (Fig. 2) included taxa that were involved either in coabundance (positive associations represented by green line) or coexclusion (negative associations represented by pink line) based on a threshold of *P < *0.05, and an absolute pairwise correlation of >0.30. Nodes were categorized as hubs or non-hubs based on the within-module degree and eigenvector centrality. Eigenvector considers both the importance of the node and the degree of connectivity of its neighbors. The hub nodes were further classified as network hubs (modularity > 0.63) and module hubs (modularity between 0.60 and 0.63) based on the degree of connectivity. Based on both eigenvector centrality and modularity, 5 centered genera (*Lactobacillus*, [*Clostridium*]*_methylpentosum_group*, Acinetobacter, *Phascolarctobacterium*, and an uncultured member from the family *Erysipelatoclostridiaceae*) were identified as hubs. This rendered the potential of identified hubs in diverse species interactions. Possible competition interactions were identified in Fig. 2, where *Bacteroides* relative abundance was negatively correlated with *Lactobacillus*, the [*Clostridium*] *methylpentosum* group, and an uncultured member of the *Erysipelatoclostridiaceae* family. In addition, genera *Alistipes* showed a positive correlation to *Bacteroides*, indicating mutualism between these genera.

Spearman correlation between cecal microbial taxa also revealed positive association between *Bacteroides* and the genera *Faecalibacterium*, *Anaerofilum*, *Anaeroplasma*, *Alistipes*, and an undetermined genus from the order *Oscillospirales*; whereas negative correlations between *Bacteroides* and the genera *Lactobacillus*, Escherichia*-Shigella*, *Blautia*, *Subdoligranulum*, *Anaerostipes*, *Negativibacillus*, the [*Ruminococcus*]-torques group, and an uncultured genus belonging to the family *Ruminococcaceaea* were also suggested (Fig. S1).

### HB individuals have higher short-chain fatty acid concentrations in cecal contents.

**FIG 3:**
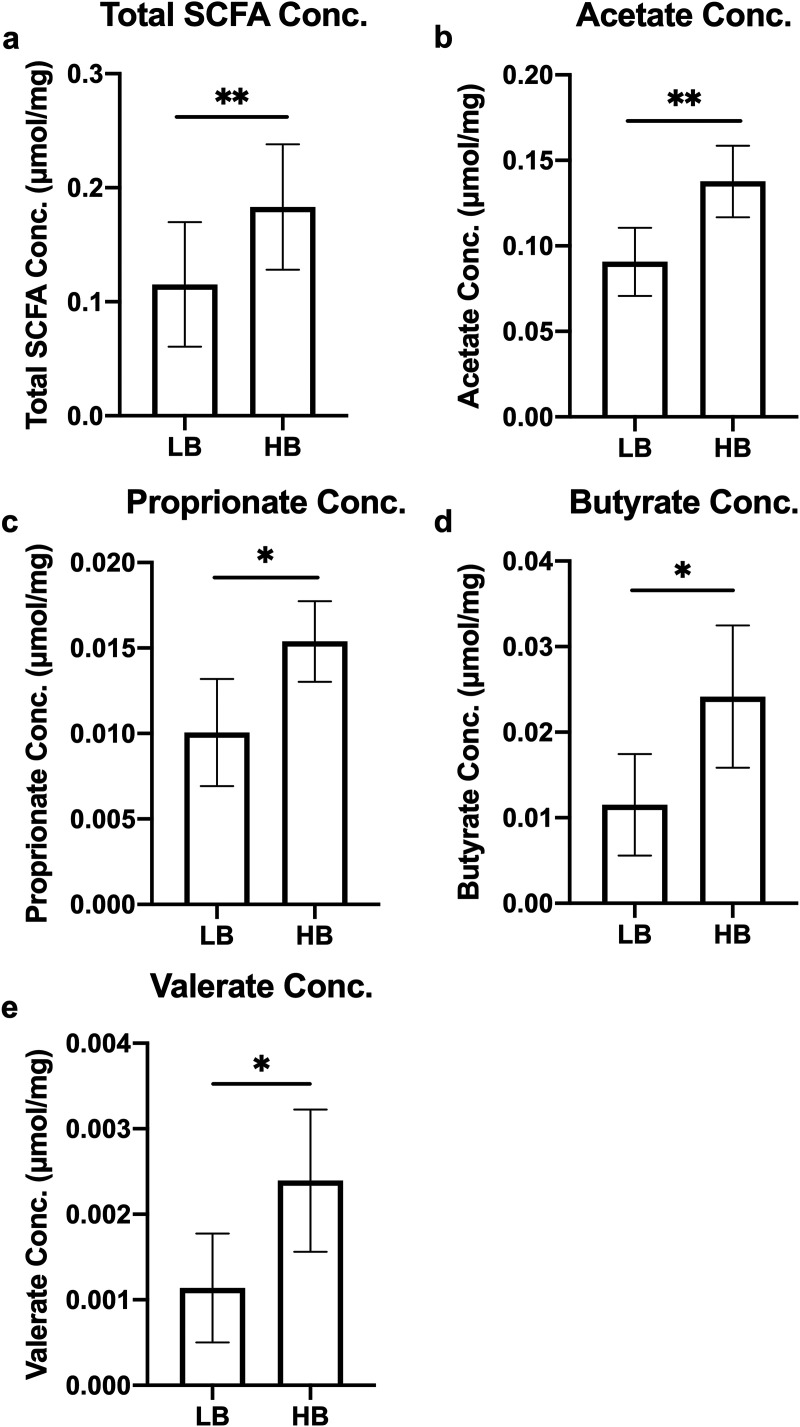
Cecal short-chain fatty acid (SCFA) concentrations in LB and HB groups. Results were shown as (a) total SCFA concentrations, (b) acetate, (c) propionate, (d) butyrate, and (e) valerate (mean ± standard deviation; LB, *n* = 18; HB, *n* =15; *, *P* < 0.05; **, *P < *0.01). LB, low *Bacteroides*; HB, high *Bacteroides*; Conc., Concentration.

**FIG 4:**
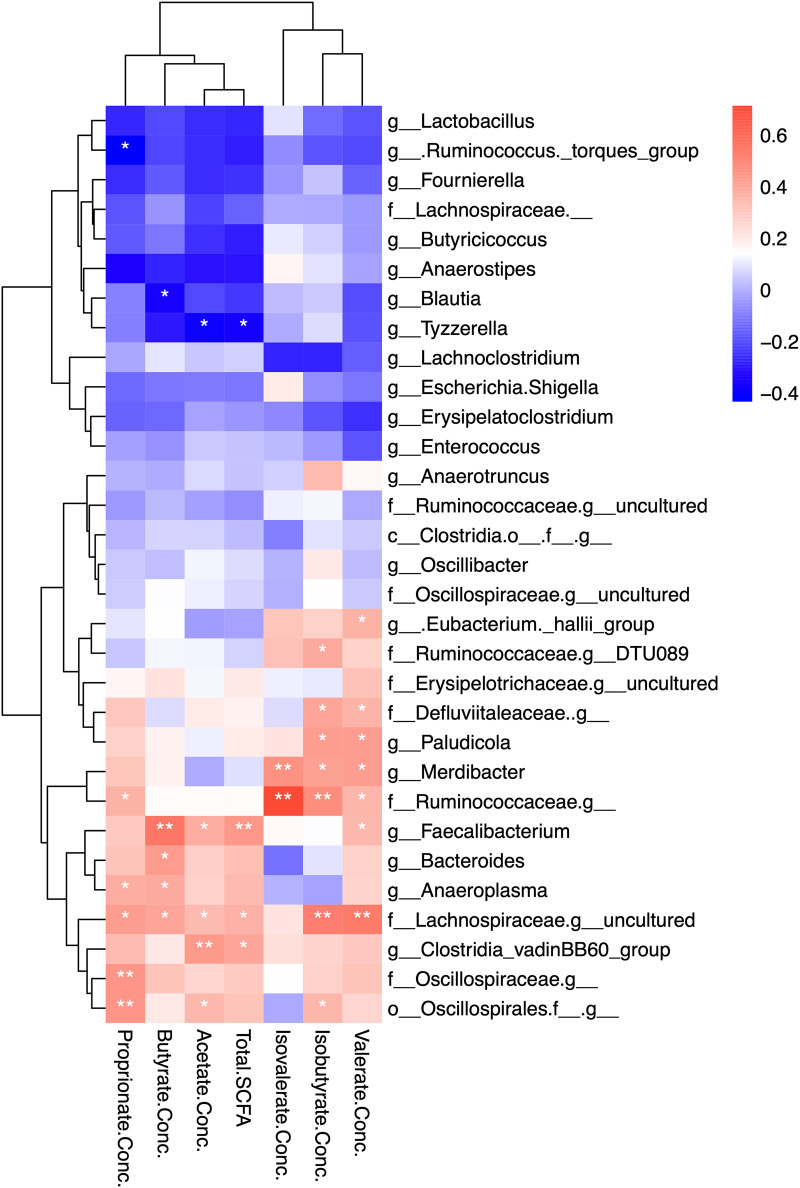
Heatmap showing Spearman correlations between cecal bacterial abundance and short-chain fatty acid (SCFA) concentrations in broiler chickens. *, *P* < 0.05; **, *P < *0.01; Conc., Concentration.

Gas chromatography was used to measure short-chain fatty acid (SCFA) concentrations in broiler cecal contents. The HB group had increased concentrations of total SCFAs (*P < *0.01), acetate (*P < *0.01), propionate (*P < *0.05), butyrate (*P < *0.05), and valerate (*P < *0.05) compared with the LB group (Fig. 3). Spearman correlation between SCFA concentrations and bacterial relative abundance suggested a series of microbes that were correlated with the altered SCFA profile between HB and LB (Fig. 4). Notably, an uncultured member belonging to the family *Lachnospiraceae* and *Faecalibacterium* were found positively associated with most of the detected SCFAs. In addition, acetate concentration was positively correlated with *Clostridia* vadinBB60 group, and negatively correlated with *Tyzzerella.* Butyrate was positively correlated with *Bacteroides*, and negatively correlated with *Blautia*. *Anaeroplasma*, a member from the order Oscillospirales, and an undetermined genus from the family *Ruminococcaceae* were associated with propionate concentration, whereas [*Ruminococcus*] torques group showed a negatively correlation with propionate levels. Branched-chain fatty acid, isobutyrate, and isovalerate, were associated with *Merdibacter*, and an undetermined member of *Ruminococcaeae.*

### Shotgun metagenomic sequencing suggested differentiated microbial functional capacities between the HB and LB group.

**FIG 5:**
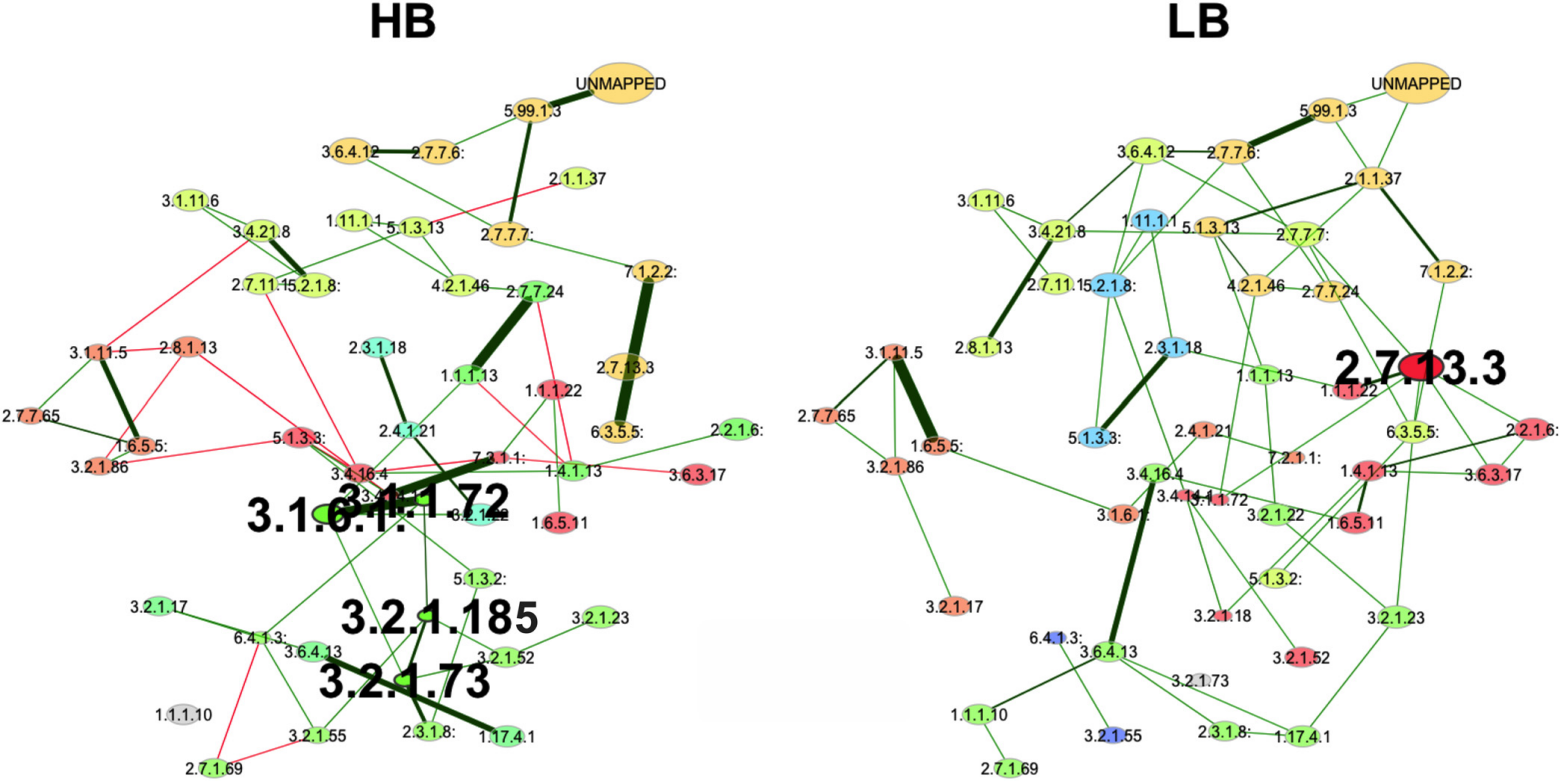
The comparison of the functional network harbored by cecal microbial communities. Green edges corresponded to positive associations, and red edges to negative associations. Colors of nodes represented clusters determined by the fast greedy modularity optimization. Node sizes were scaled according to eigenvector centrality. Nodes with bold text were identified hubs in the networks. Hubs were nodes with eigenvector centrality greater than 90% quantile of the empirical centrality distribution (LB, low *Bacteroides*; HB, high *Bacteroides*; LB, *n* = 18; HB, *n* = 15).

To investigate functional capacities of the HB and LB gut microbiome, we performed shotgun metagenomic sequencing. Briefly, a total of 1,860 genes were annotated based on the Metacyc database (12). Gene networks were constructed based on the annotated genes from shotgun metagenomic sequencing. Genes that represented characteristics of the HB group and the LB group were predicted based on eigenvector centrality (Fig. 5). The properties of the networks can be found in Table 2, 3, and 4, and Table S1. The Jaccard index was significantly close to 0 for betweenness centrality, closeness centrality, and eigenvector centrality (Table 4), suggesting that the sets of most central were considerably different between the HB and LB group (Jaccard index ranging from 0 to 1, where 0 being 2 completely different sets, and 1 being exactly equal sets). Different hub nodes were identified between HB and LB group based on eigenvector centrality (Fig. 5) that indicated nodes not only important by itself, but also sharing high connectivity with important neighbors. Specifically, in the HB network the acetylxylan esterase (EC3.1.1.72), the type I arylsulfatase (EC3.1.6.1), the non-reducing end beta-L-arabinofuranosidase (EC3.2.1.185), and the licheninase (EC3.2.1.73) were identified as hubs, whereas only the histidine kinase (EC2.7.13.3) was identified as a hub in the LB group.

**TABLE 2:** Properties of networks constructed for the HB group and LB group, Jaccard index^*a*^

Network properties	Jacc	*P* (≤Jacc)	*P* (≥Jacc)
Degree	0.048	0.002**	1.000
Betweenness centrality	0.000	< 0.001***	1.000
Closeness centrality	0.091	0.009**	0.998
Eigenvector centrality	0.050	0.003**	1.000
Hub taxa	0.000	0.296	1.000
Adjusted rand index = 0.109 (ARI, measuring similarity between clusterings ranging from 0–1. ARI = 1, perfect agreement between clusterings; ARI = 0, two random clusterings; *P* <0.001)			

a Jaccard index measured the similarity between sets of most central nodes. Jaccard index ranged from 0 (completely different) to 1 (sets equal), **, *P* < 0.01; ***, *P* < 0.001.

**TABLE 3:** Properties of networks constructed for the HB group and LB group, Hub nodes

HB	LB
3.1.1.72: Acetylxylan esterase	2.7.13.3: Histidine kinase
3.1.6.1: Arylsulfatase (type I)	
3.2.1.185: Non-reducing end beta-L-arabinofuranosidase	
3.2.1.73: Licheninase	

**TABLE 4:** Eigenvector centrality

Gene	HB	LB
Highest values in the HB group		
3.2.1.185: Non-reducing end beta-L-arabinofuranosidase	1.000	0.267
3.2.1.73: Licheninase	0.949	0
3.1.1.72: Acetylxylan esterase	0.866	0.558
3.1.6.1: Arylsulfatase (type I)	0.839	0
3.2.1.52: Beta-N-acetylhexosaminidase	0.665	0.132
Highest values in the LB group		
2.7.13.3 histidine kinase	0	1.000
2.7.7.7: DNA-directed DNA polymerase	0	0.912
6.3.5.5: Carbamoyl-phosphate synthase (glutamine-hydrolyzing)	0	0.752
2.7.7.6: DNA-directed RNA polymerase	0	0.737

### Shotgun metagenomic sequencing suggested enriched microbial pathways related to complex carbohydrate degradation and SCFA production in the HB group.

**FIG 6:**
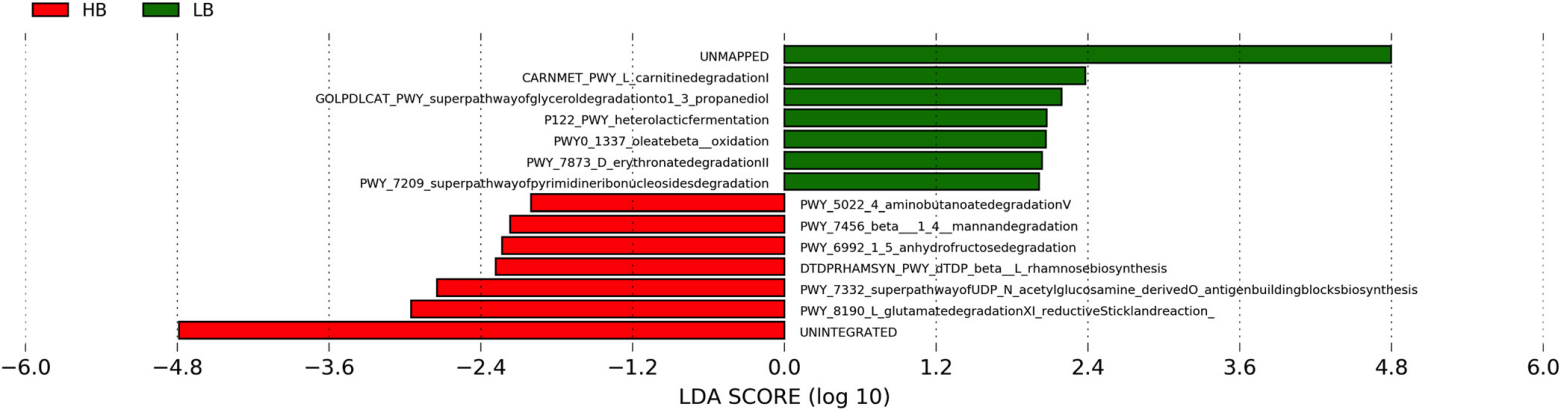
Linear discriminant analysis (LDA) effect size (LEfSe) showing differentially abundant pathways of the cecal microbiota (LDA score ≥ 2.0; *P < *0.05; LB, low *Bacteroides*; HB, high *Bacteroides*; LB, *n* = 18; HB, *n* = 15).

Shotgun metagenomic sequencing and functional genomics analyses identified 12 pathways that were different between the HB group and the LB group (LefSe LDA score > 2) (Fig. 6). The gut microbiota of the HB group harbored more abundant pathways, including the Stickland reaction pathways (PWY-8190), the superpathway of UDP-*N*-acetylglucosamine-derived O-antigen building blocks biosynthesis (PWY-7332), the dTDP-β-L-rhamnose biosynthesis (DTDPRHAMSYN-PWY), the 1,5-anhydrofructose degradation pathway (PWY-6992), the β-(1, 4)-mannan degradation pathway (PWY-7456), and the γ-aminobutyric acid degradation pathway (PWY-5022). The LB microbiota were more abundant in the l-carnitine respiration pathway (CARNMET-PWY), the superpathway of glycerol degradation to 1,3-propanediol (GOLPDLCAT-PWY), the heterolactic fermentation pathway (P122-PWY), the oleate β-oxidation pathway (PWY0-1337), the d-erythronate degradation II pathway (PWY-7873), and the superpathway of pyrimidine ribonucleosides degradation pathway (PWY-7209), which exerts reductive pyrimidine degradation in bacteria.

### The cecal tonsil of HB and LB chickens exhibited different expression levels of genes involved in immune tolerance and gut integrity.

**FIG 7:**
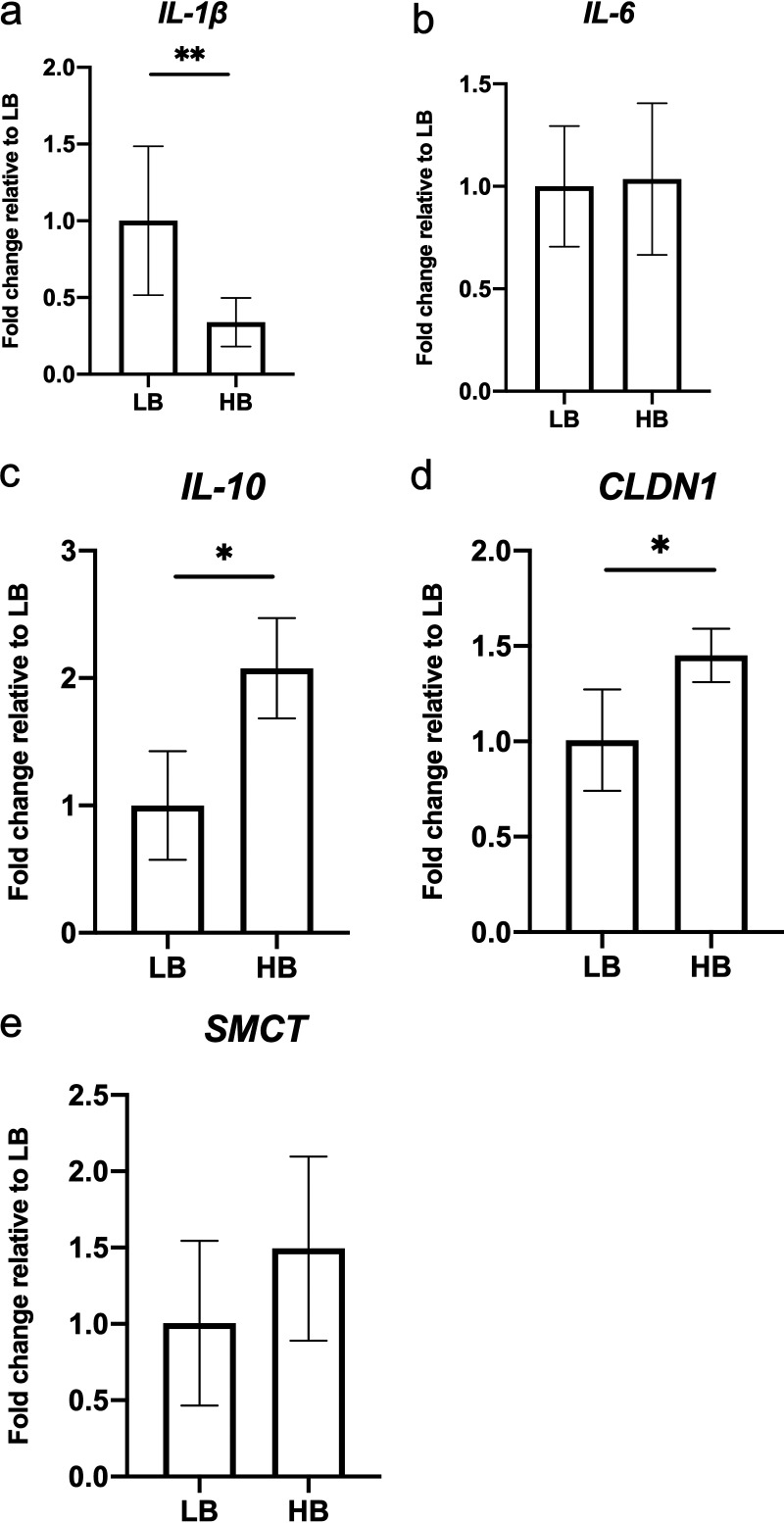
Gene expression in chicken cecal tonsil. (a) Pro-inflammatory cytokine *IL-1β* showed lower expression levels in the HB group compared to LB broiler chickens. (b) *IL-6* expression was not differed between groups. (c) Increased anti-inflammatory cytokine *IL-10* was observed in the HB group. (d) The expression of the tight-junction protein *CLDN1* was slightly increased in the HB group. (e) Compared to the LB group, a trend of increased expression of short-chain fatty acid transporter *SMCT* was seen in the ceca of HB chickens (LB, *n* = 18; HB, *n* = 15; mean ± standard deviation; *, *P < *0.05; **, *P < *0.01; n.s., not significant; LB, low *Bacteroides*; HB, high *Bacteroides*).

To investigate how different levels of *Bacteroides* population affected host responses, cecal tonsil RNA was extracted from the HB and LB group, and subjected to reverse transcription qPCR (RT-qPCR) assay to examine immune-related genes, including Interleukin (*IL*)*-1β*, *IL-6* and *IL-10*, as well as tight-junction protein gene *claudin-1* (*CLDN1*), and the sodium coupled monocarboxylate transporter (*SMCT*) (Fig. 7). The cecal tonsils of the HB group showed a decreased *IL-1β* with an increased *IL-10*, compared with the LB group. In addition, *CLDN1*, which has previously been associated with improved barrier function (13), was also higher in the cecal tonsil tissues of the HB group (*P < *0.05). *SMCT* expression was not different between groups (*P = *0.103).

## DISCUSSION

While several studies have shown significant variations in *Bacteroides* populations in the chicken gut microbiome in early life (6, 7), this is the first study to investigate how distinct *Bacteroides* compositions associate with cecal SCFA profiles, host responses, as well as microbial functional capacity. Bodyweight of day-7 chickens were not collected, limiting a connection to growth performance. However, 32-day bodyweight and flock mortality rate were similar between flocks, where the majority of birds were identified as LB, n/a, or HB. With regard to the cecal microbial composition of chickens close to the end of production, the variability of *Bacteroides* was much less pronounced (data not shown); therefore, the LB/HB phenotype was only explored in day-7 chickens. Moreover, in this study, only 5 young broilers per flock were sampled. Previously, microbiome studies showed that individuals housed together, particularly coprophagic animals like mice and chicken, exhibited high similarity in the intestinal microbiota (14, 15). Therefore, to better explore the variability of the cecal microbiome among commercial broiler chickens, instead of sampling more broilers from each flock, we chose to increase the number of flocks sampled.

What caused the distinct *Bacteroides* levels was not investigated in thisstudy. Previous studies showed that cecal *Bacteroides* abundance in young broiler chickens could be increased by exposure to cecal contents from 40-week-old healthy chickens via oral gavage (16), the use of recycled litter (17), or hen contact at hatching (4). The absence of contact with the parent flock in modern broiler production likely limits the transmission of mature chicken-derived commensals (2, 18). In this sense, the alternate initial exposures (e.g., parent flock, hatching environments, or handling crew) may play an important role in shaping the early-life broiler microbiota. It has been reported that the relative abundance of *Bacteroides* was positively correlated with chicken cecal SCFA profiles (19). In accordance with previous findings, our results showed that the over-representation of *Bacteroides* in ceca was associated with increased concentrations of SCFAs, particularly acetate, propionate, butyrate, and valerate. In the chicken intestine, SCFAs are products of the gut microbiota fermentation from partially- or non-digestible polysaccharides, mainly derived from plant biomass. Functional gene network analyses showed that the microbial functional capacity of the HB group was centered by a series of complex carbohydrate degradation enzymes. Specifically, acetylxylan esterase (EC3.1.1.72) contributes to xylan utilization (20), and β-L-arabinofuranosidase (EC3.2.1.185) helps digest glycoproteins that are widely found in plant cell wall fractions (21). The licheninase (EC3.2.1.73) can degrade β-glucans, which have been used as chicken feed additives (22), and were found to modulate the host gut microbiota, decreasing intestinal inflammation (23, 24). In this study, Spearman correlation analyses showed that the abundance of the microbe-encoded acetylxylan esterase, β-L-arabinofuranosidase, and licheninase were significantly associated with cecal total SCFA, acetate, propionate, butyrate, and valerate concentrations (Fig. S2). Therefore, by harboring microbial functional capacity centered by these key enzymes, the microbiota of the HB group showed potential for increased utilization of plant-derived biomass to promote SCFA production, and thereby improve gut health.

Van der Hee and Wells (2021) recently reviewed the complex interactions between SCFAs, gut microbes, and the host immune system (25). Briefly, enterocytes can absorb SCFAs via passive diffusion or protein-mediated transport, and elevated levels of lumen SCFAs enhance associated transporter and receptor expression (25). Nastasi et al. (2015) reported that butyrate can confer anti-inflammatory properties in colonic dendritic cells via the G-protein coupled receptors pathway, which inhibits the expression of cytokine and chemokine genes (26). In this study, the elevated butyrate in HB birds coincided with lower *IL-1β*, and higher *IL-10* expression in the cecal tonsil. In addition, tight-junction protein levels are important indicators of gut integrity, as they contribute to epithelial cell adhesions and act as a barrier. Generally, decreased expression of tight-junction proteins may lead to diffusion of antigens or bacterial macromolecules (e.g., endotoxin) from the intestinal lumen into circulation (27). Decreased level of tight-junction protein claudin 1 was reported in chronically stressed, and repeatedly corticosterone-injected rats (28). In addition, gut inflammation caused by Salmonella enterica serovar Typhimurium challenge was also found to decrease the expression of chicken intestinal claudin 1 (13). Therefore, in this study, the decreased expression of *CLDN1* mRNA level found in the LB group may indicate decreased gut integrity, and may have contributed to the increased expression of *IL-1β*.

Microbial co-occurrence networks provided an opportunity to explore the impact of elevated *Bacteroides* on cecal microbial communities and types of interactions with other connected microorganisms. The analysis included both positive and negative links, considering the possibility that both types of associations could influence network stability. To circumvent the limitations of sparsity and high dimensionality of microbial data, the correlation principle was utilized to understand the pairwise associations among microbes and interactions. Further, network features were computed to identify biologically significant patterns and community keystone taxa. In this study, the SparCC correlation method evaluated the variance of the log-ratio for modified data to infer pairwise relations. *Lactobacillus* was negatively associated with *Bacteroides* in the cecal microbial community. Similarly, previous human studies have demonstrated that *Lactobacillus* can competitively exclude commensals, including *Bacteroides* (29). Competition is often observed between taxa sharing similar nutrient sources (e.g., nitrogen and carbon source). It might partially explain the negative correlations between *Bacteroides* and *Lactobacillus* in this study, since members from these 2 genera are efficient and important complex carbohydrate degraders. Particularly, our results of functional genetic analyses indicated that the 1,5-anhydrofructose degradation pathway (PWY-6992), and the β-(1, 4)-mannan degradation pathway (PWY-7456) were more abundant in the HB cecal microbiota. The 1,5-anhydrofructose degradation pathway catalyzes the degradation of glycogen (30), whereas the β-(1, 4)-mannan degradation pathway is involved in the hydrolysis of mannans, a major group of hemicellulose (31). The enriched pathway PWY-7456 in the HB cecal microbiota indicated that the microbial members harbored greater genetic potential in utilizing complex carbohydrates derived from plant cell wall, which were contained in chicken feed. In the LB group, the heterolactic fermentation pathway (P122-PWY) was more abundant in the gut microbiome. Possessed mainly by lactic acid bacteria, the heterolactic fermentation pathway ferments starch to lactates. The difference in the predominant carbohydrate utilization pathways between LB and HB groups further identified nutrient competition between *Bacteroides* and *Lactobacillus*, particularly regarding complex carbohydrate fermentation. Currently, a good number of studies have considered *Lactobacillus* as probiotics in poultry, and reported potential beneficial effects. However, many of these studies also found that the abundance of *Lactobacillus* in the chicken ceca was not affected by *Lactobacillus* supplementation, suggesting that the potential beneficial effects conferred by *Lactobacillus* may not be a consequence of cecal colonization (32, 33). In fact, Chen et al. (2016) studied the effect of the supplementation of *Lactobacillus* spp. and/or yeast with bacteriocin on broiler performance, and reported that supplementation with *Lactobacillus* culture alone (without bacteriocin) was the only treatment group that increased cecal *Lactobacillus* colonization (34). Consistent with our study, the increase in *Lactobacillus* coincided with decreased SCFA production, with no improvement on performance compared to the control (34). Thus, although supplementing *Lactobacillus* had been shown to exert beneficial effects on poultry, the effects of *Lactobacillus* colonization in the chicken ceca needs to be carefully assessed in the future.

In addition, results from this study indicated that *Alistipes* may share a mutualistic relationship with *Bacteroides.* To date, little is known about the genus *Alistipes*, which is a sub-branch genus of the phylum Bacteroidetes (35). *Alistipes* are bile-resistant microorganisms with an ability to produce acetic acid by digesting gelatin and fermenting carbohydrates (36). Correlations between health outcomes and *Alistipes* indicated that *Alistipes* may exert protective effects against diseases, such as liver fibrosis and colitis (37, 38). However, the causal effect of the microbial taxa in diseases and its prevalence remains unclear. Studies have shown that *Alistipes* are more abundant in host gut with an anti-inflammatory background (39, 40). In this study, the lower *IL-1β* expression in the ceca of HB broilers may have favored *Alistipes* growth.

The higher *Bacteroides* abundance in ceca may reflect a further progression of microbial succession, with the transition from facultative anaerobes, such as *Lactobacilli*, to strict anaerobes, such as *Bacteroides*, *Ruminococcaceae*, and *Lachnospiraceae*. An anaerobic gut environment and undigested carbohydrates entering ceca are essential for the production of SCFAs (41), in turn, increased SCFAs help make the gut more anaerobic (42). In congruence, our results showed that obligate anaerobes from the families *Ruminococcaceae* and *Lachnospiraceae* were positively correlated with SCFA production. On the other hand, more inflammation could provide reactive oxygen species that could delay colonization of strict anaerobes (43). Although previously both *in vitro* (44) and *in vivo* (45) studies have shown the immunomodulatory effects of *Bacteroides* strains, the direction of causality between high *Bacteroides*/SCFA levels and inflammation has yet to be determined. Therefore, future studies assessing how the host intestine responds to increased SCFA (e.g., via histone deacetylation) is warranted. Further, while this study points to a beneficial impact of high *Bacteroides* colonization, future research with the introduction of *Bacteroides* strains to chickens in microbially controlled conditions will be needed to demonstrate causal contributions of *Bacteroides* in improving health outcomes, and to support their development as potential probiotics.

To conclude, this study identified distinct *Bacteroides* populations in the ceca of commercial broiler chickens in early life. Our results revealed that elevated level of cecal *Bacteroides* in young chickens had led to altered microbial functional capacity of the gut microbiome, which promoted the production of SCFA. Coinciding with that, compared to the LB group, chickens from the HB group had lower expression of pro-inflammatory cytokines, coupled with higher expressions of anti-inflammatory cytokine and tight-junction protein gene. Consequently, it indicated that elevated cecal *Bacteroides* may be beneficial to commercial broiler chickens in suppressing gut inflammation through the increment of short-chain fatty acid production.

## MATERIALS AND METHODS

### Chicken management and sample collection.

Following the Canadian Council on Animal Care guidelines (46), the animal usage of this experiment was approved by the Animal Care and Use Committee administered by the University of Alberta (AUP00002377). A commercial broiler farm in AB, Canada provided facilities and all the chickens for this study. A total of 14 broiler flocks reared under the same feed and water, light exposure, and immunization condition in similarly engineered broiler production houses were sampled. Animal management and sample collection procedure were performed as descried previously (47). Briefly, for each flock, 14,000 Ross 308 broiler chicks were placed at 1 day of age, and fed *ad libitum* until the end of the production cycle. At day 7, five broiler chickens from each flock, randomly selected from different areas in the barn, were euthanized by cervical dislocation for sampling. Approximately 300 mg of cecal contents and cecal tonsil tissue were collected, snap-frozen, and stored at –80°C for further analyses.

### *Bacteroides* over-/under- representing sample identification.

Total DNA was extracted from cecal contents using the QIAamp Fast DNA Stool minikit (Qiagen), with an additional bead-beading step with ~200 mg of garnet rock at 6.0 m/s for 60 s (FastPrep-24 5G instrument, MP Biomedicals). Amplicon libraries were constructed according to the manufacturing protocol from Illumina (16S Metagenomic Sequencing Library Preparation), targeting the V3-V4 region of the 16S rRNA gene (primers: Forward: 5′-TCGTCGG CAGCGTCAGATGTGTATAAGAGACAGCCTACGGGNGGCWGCAG-3′; Reverse: 5′-GTCTCGTGGGCTCGGAGATGTGTATAAGAGACAGGACTACHVGGGTATCTAATCC-3′). An Illumina MiSeq Platform (2 × 300 cycles; Illumina Inc.) was used for a paired-end sequencing run.

The quality of the reads was assessed using FastQC. Quantitative Insight into Microbial Ecology (QIIME2)-2020.6 was used to process the sequenced reads (48). DADA2 was used to de-noise and generate paired-end representative reads (49), and samples with reads less than 14,000 reads were removed. An ASV feature table was subsequently created. To assign taxonomy, the q2-feature-classifier in QIIME2 was used with a pretrained classifier “SILVA 132 99%” (50). The command “qiime feature-table relative-frequency” on taxa collapse level 6 was used to calculate genus relative abundance in QIIME2. The mean value and standard deviation of *Bacteroides* relative abundance was calculated. Based on the data distribution, to screen for distinct cecal *Bacteroides* samples, cecal content with *Bacteroides* relative abundance falling inside one standard deviation were considered as non-assigned (n/a) samples, whereas the ones falling outside of a standard deviation was considered as LB or HB samples.

The 'diversity core-metrics-phylogenetic' command was used for diversity analyses on the screened samples. The Chao1 and Shannon diversity indices were computed using “diversity alpha-phylogenetic”, and the significance was determined using “diversity alpha-group-significance”. The beta diversity was analyzed in QIIME2 using the Bray-Curtis distance metric, and a principal coordinate analysis (PCoA) was plotted in R utilizing phyloseq package. Pairwise Permutational Multivariate Analysis of Variance Using Distance Matrices (pairwise Adonis) based on the Bray-Curtis distance matrices was used to identify significant differences in community structures between treatments.

A microbial co-occurrence network was calculated using the NetCoMi package (version 1.0.2) in R, with the Sparse Correlations for Compositional data (SparCC) as the sparsification method. The algorithm estimated pairwise association after 20 iterations, assuming an absence of a large number of co-occurring taxa with strong correlations. The taxa count data was resampled 100 times before being used to generate randomized correlation tables. For each pairwise correlation, the randomized correlation matrix was used to calculate bootstrapped *P* values. The resulting correlation matrix was utilized in network models to define links between taxa. If the absolute pairwise correlation between 2 taxa was greater than 0.25, and there was strong evidence for the association (*P < *0.001), correlations between the 2 taxa were considered during network construction. Network features, including degree, betweenness, closeness centrality, and modularity computation, enabled identification of hubs (quantile set at 0.9). The community structure was constructed based on the fast, greedy algorithm (51).

The extracted genomic DNA was also used to measure the abundance of the *Bacteroides-Prevotella* group in the cecal content using qPCR, targeting the 16s rRNA gene (Table 5). PerfeCTa SYBR green Supermix (Quantabio) was used for qPCR assays, which were conducted on an ABI StepOne real-time system (Applied Biosystems), following the setup of 95°C for 3 min, 40 cycles of 95°C for 10 s, and 68°C for 30 s. A 10-log-fold standard curve for quantification of the target gene was created using PCR amplicon, where the concentration was determined by a Quant-iT PicoGreen dsDNA assay kit (Invitrogen). *Bacteroides-Prevotella* 16S rRNA gene copy numbers were determined using the relative standard curve method, and normalized to the weight of cecal content used for DNA extraction.

**Table 5:** Primers used in this study

Primer	Sequence	Product size (bp)	Reference
*Bacteroides-Prevotella* Forward	GGTGTCGGCTTAAGTGCCAT	140	56
*Bacteroides-Prevotella* Reverse	CGGACGTAAGGGCCGTGC		
IL^*a*^-1b Forward	GGGCATCAAGGGCTACAA	88	57
IL-1b Reverse	CTGTCCAGGCGGTAGAAGAT		
IL-6 Forward	GAGGGCCGTTCGCTATTTG	67	58
IL-6 Reverse	ATTGTGCCCGAACTAAAACATTC		
IL-10 Forward	GCTGAGGGTGAAGTTTGAGG	121	59
IL-10 Reverse	AGACTGGCAGCCAAAGGTC		
SMCT^*b*^ Forward	GGCTTCAGCGTTTGGGACTA	235	60
SMCT Reverse	TGCAGAAGATGGCACCGTAG		
CLDN^*c*^1 Forward	CCAGGTGAAGAAGATGCGGA	129	
CLDN1 Reverse	GGTGTGAAAGGGTCATAGAAGGC		
GAPDH^*d*^ Forward	CTACACACGGACACTTCAAG	244	27
GAPDH Reverse	ACAAACATGGGGGCATCAG		

a IL, interleukin.

b SMCT, sodium coupled monocarboxylate transporter.

c CLDN, claudin.

d GAPDH, Glyceraldehyde 3-phosphate dehydrogenase.

### Shotgun metagenomic sequencing and functional genomics analyses.

Total genomic DNA extracted from the cecal contents, as described above, were used for Shotgun metagenomic sequencing. Library preparation and shotgun sequencing were performed at the Genome Quebec Innovation Centre (Montreal, Canada). Libraries were generated using NEBNext Ultra II DNA Library Prep Kit (New England Biolabs). Shotgun metagenomic sequencing was performed using the NovaSeq 6000 S4 PE150 system (Illumina Inc.).

FastP v0.23.2. was used for quality control. Low quality reads, adaptors, polyG, and duplicated sequences were removed (52). To remove host DNA contamination, a chicken host reference database was built using bowtie2 v2.4.1 with genome *Gallus_gallus* 105 release from Ensembl (53). Kneaddata v0.10.0 were used to remove host contaminants with the built reference database (https://github.com/biobakery/kneaddata). Gene abundance and pathway analyses were conducted using HuMAnN3, with default settings (54). Gene and pathway abundance were annotated by the Metacyc database, and normalized to copy numbers per million reads using the HuMAnN3 utility scripts. Differentiate gene and pathway abundance were identified using LDA effect size (LEfSe) implemented in the Galaxy online tool (LDA score > 2). Differentiate gene network was constructed using the NetCoMi v1.0.2 package in R (51). Briefly, filter parameters were set to the 50 most frequent genes. Gene network was clustered based on the fast, greedy algorithm. Gene association was determined by the SPRING method (55). The corresponding similarities were used as edge weights. Eigenvector centrality was used to define hubs and scale node sizes. Properties of the constructed network were calculated based on the highest degree, and betweenness and closeness centrality at the same time, with hub quantile set at 0.9. The Jaccard index was used to assess the differences of the most central nodes between groups. Similarity between networks were assessed based on the adjusted Rand index.

### RT-qPCR assay.

To examine host response to different *Bacteroides* relative abundance, cecal tonsils were subjected to RNA extraction, followed by cDNA synthesis and qPCR assay. Approximately 30 to 50 mg of snap-frozen cecal tonsil tissue was cut and weighed. Tissue was ground by pre-chilled RNase-free mortar and pestle in liquid nitrogen. RNA was extracted using the GeneJET RNA purification kit (Thermo Scientific) with modifications. Specifically, ground tissues were homogenized in 600 μL of lysis buffer followed by bead beating in nuclease-free tubes with 3 metal beads at 4 m/s for 20 s (MP Biomedicals). Prior to elution, DNase I (Qiagen) was used to treat samples for 15 min to remove DNA. RNA concentration was determined by a NanoDrop 2000c spectrophotometer (Thermo Scientific), and were normalized to 1 μg of RNA for reverse transcription. QScript Flex cDNA Synthesis Kit (Quanta Biosciences) was used for RNA reverse transcription, following the random primer and oligo (dt) protocol. Then, qPCR was performed using PercfeCTa SYBR green Supermix (Quantabio) with primers listed in Table 5, and conducted on an ABI StepOne real-time system following the cycles: 95°C for 3 min, 40 cycles of 95°C for 10 s, and 60°C for 30 s. Glyceraldhyde-3-phosphate dehydrogenase (GAPDH) was used as the housekeeping gene for calculating the fold change of gene expression relative to LB birds using the 2^-ΔΔCt^ method.

### SCFAs analysis.

Cecal contents used for 16S rRNA gene amplicon sequencing, and shotgun metagenomic sequencing were also used for SCFA analysis. Approximately 30 mg per sample of snap-frozen cecal content was weighed, followed by homogenization with 25% phosphoric acid. Samples were centrifuged at 21,130 × *g* for 10 min, and supernatant was collected and filtered using a 0.45 μm filter. Isocaproic acid (23 μmol/mL) was added at a 1:4 ratio to samples as an internal standard. Samples were analyzed on a Scion 456-GC instrument. Final concentrations of SCFAs were normalized to sample weights.

### Statistical analyses.

If not otherwise stated, statistical analyses were conducted using GraphPad Prism 8 (GraphPad Software), and mean values were presented as mean ± the standard deviation. Statistically significant differences were determined (*P < *0.05) by an unpaired Student's *t* test for parametric data (i.e., gene expression and SCFA concentrations). The Kruskal–Wallis test was used to determine the significance of non-parametric data (i.e., microbiome alpha-diversity indices). The Spearman’s correlation was used to correlate SCFA concentration and bacterial relative abundance, as well as to determine correlations between cecal microbial taxa. Correlation significance was determined by psych package, and visualized using the corrplot package in R (version 3.6.1).

### Data availability.

The 16S rRNA sequences and shotgun metagenomic sequences in this study were submitted to NCBI Sequence Read Archive under BioProject IDs: PRJNA876288 and PRJNA902117, respectively.
