# Supplementary material for: Week-Old Chicks with High Bacteroides Abundance Have Increased Short-Chain Fatty Acids and Reduced Markers of Gut Inflammation
Source: Microbiol Spectr. 2023 Jan 31;11(2):e03616-22. doi: 10.1128/spectrum.03616-22 (PMC10100795; doi:10.1128/spectrum.03616-22)
Supplement: Supplemental file 1 — Fig. S1 and S2. Download spectrum.03616-22-s0001.pdf, PDF file, 0.6 MB [file spectrum.03616-22-s0001.pdf]

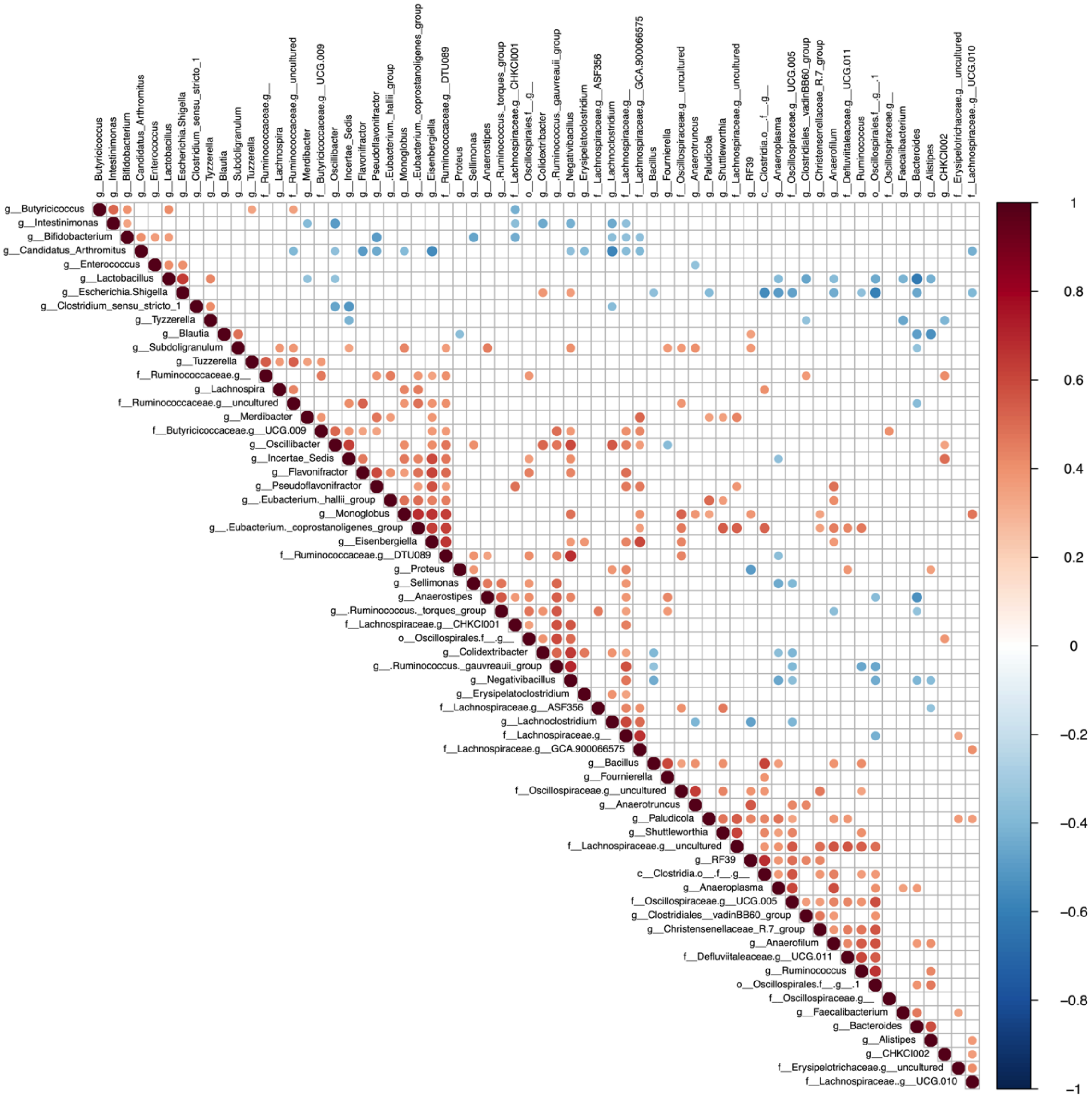


Figure S1. Spearman's correlation between cecal microbial taxa. Only significantly correlated taxa (P < 0.05) were shown. Spearman correlations between microbes revealed that *Bacteroides* was negatively correlated with the genera *Lactobacillus*, *Escherichia*-*Shigella*, *Blautia*, *Subdoligranulum*, *Anaerostipes*, *Negativibacillus*, the *[Ruminococcus]-torques*-group, and an uncultured genus belonging to the family *Ruminococcaceaea*. In addition, the genera *Faecalibacterium*, *Anaerofilum*, *Anaeroplasma*, *Alistpe*, as well as an undetermined genus from the order *Oscillospirales* showed positive correlations to *Bacteroides*.

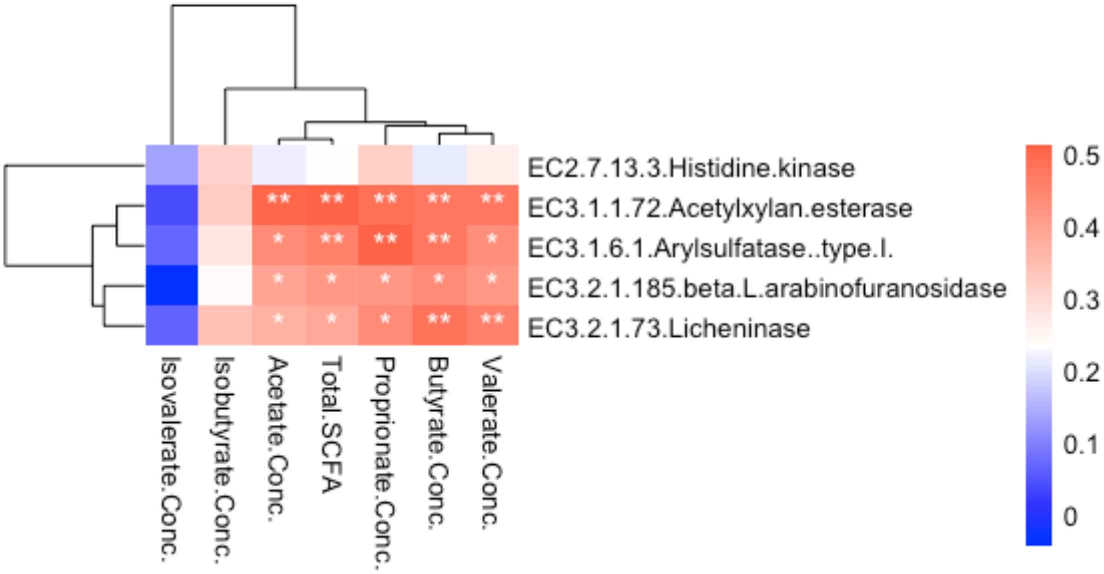


Figure S2. Spearman's correlation between the key enzymes of the chicken cecal microbial functional network and cecal SCFA concentrations. Key enzymes identified in the microbial functional network of the microbiome with elevated *Bacteroides* (i.e. acetylxylan esterase, arylsulfatase, β-L-arabinofuranosidase and licheninase) were significantly associated with the chicken cecal SCFA concentrations at day 7.
